# Residual feed intake in laying hens during the late laying period: associations with nutrient utilization, antioxidant capacity, and gut barrier function

**DOI:** 10.1186/s40104-026-01426-7

**Published:** 2026-07-28

**Authors:** Zhouyang Gao, Zhiqiong Mao, Qiujuan Sun, Chuanwei Zheng, Guoming Ma, Jingjie Zhuang, Yan Wu, Yuhui Qin, Tong Xu, Guiyun Xu

**Affiliations:** 1 College of Animal Science and Technology, China Agricultural University, Beijing, 100193 China; 2 Beinongda Technology Co., Ltd., Beijing, 100083 China; 3 Guangdong Yihao Foodstuff Co., Ltd., Guangzhou, 510620 China; 4 College of Animal Science and Veterinary Medicine, Shenyang Agricultural University, Shenyang, 110866 China

**Keywords:** Biological breeding, Late laying period, Laying hen, Metabolomics, Residual feed intake

## Abstract

**Background:**

Residual feed intake is defined as the difference between the actual feed intake of poultry and the predicted feed intake based on their growth and maintenance requirements. It reflects the metabolic differences that are genetically determined within the poultry and can effectively indicate the energy levels needed for growth and maintenance. Residual feed intake is a crucial indicator for evaluating the feed utilization efficiency of poultry. Additionally, residual feed intake is a trait with moderate to high heritability, making it a valuable selection criterion for feed efficiency in poultry breeding programs, and it is widely utilized in egg production. However, research on residual feed intake during the latter stages of egg production in hens remains quite limited.

**Results:**

In this study, we selected laying hens with similar body weight and egg production number, and divided the experiment into low residual feed intake and high residual feed intake groups based on the magnitude of residual feed intake values. Through physicochemical analysis, compared to the high residual feed intake group, the low residual feed intake group exhibited higher nutrient utilization (crude protein, apparent digestibility of dry matter) and stronger antioxidant enzyme activity (T-AOC), as well as more stable gut immune barriers (*IL-6*) and mechanical barriers (*Occludin*, *ZO-1*). LC–MS revealed that the differentially abundant metabolites in the serum and excreta of the two groups were primarily enriched in amino acid metabolism, lipid metabolism, and energy metabolism pathways (L-lysine, L-glutamine, citrate, fumarate, propanoic acid, glycine, pyruvate.). These key differentially abundant metabolites provide new metabolic targets for a deeper understanding and improvement of feed efficiency in laying hens.

**Conclusion:**

This study utilized traditional nutritional methods and modern molecular biology techniques to target the excreta, serum, and intestinal segments of hens with different RFI levels, providing an in-depth exploration of the metabolic differences between high- and low-RFI hens. The findings offer valuable insights for selecting high-efficiency, high-quality healthy laying hens through RFI. Furthermore, they support the extension of the laying period in hens and efficient breeding practices.

**Supplementary Information:**

The online version contains supplementary material available at 10.1186/s40104-026-01426-7.

## Introduction

In recent years, to better utilize poultry husbandry equipment and reduce production costs, the breeding cycle of commercial laying hens has been extended from 72 weeks [1]. However, with the prolonged breeding cycle, particularly in the latter stages of egg production, common issues such as reduced egg production rates, decreased feed utilization efficiency, and increased metabolic burdens arise [2, 3]. Therefore, improving feed utilization efficiency in the later stages of egg production is crucial for the overall growth of the poultry industry. In production practice, residual feed intake (RFI) is a core indicator for measuring feed efficiency and has been widely applied in poultry breeding.

RFI is the difference between the actual feed intake of livestock and poultry and the expected feed intake based on their body size and growth rate [4]. Dadfar et al. [5]introduced the concept of RFI to egg-type chickens; furthermore, RFI is a negatively selected trait, meaning that chickens with lower RFI values exhibit greater feed efficiency, whereas those with higher RFI values show lower feed efficiency. Moreover, RFI is independent of economic traits. In ducks, selecting for low RFI does not adversely affect egg quality and can improve feed utilization efficiency [6]. In the selection of laying hens and broilers, low RFI groups exhibit higher annual egg production, fertilization rates, and hatchability [7]. Research indicates that RFI in laying hens has moderate heritability and is a complex trait regulated by multiple genes. Basso et al. [8] studied 384 ducks and found the heritabilities of RFI, feed intake (FI), body weight (BW), and weight gain (BWG) to be 0.24, 0.34, 0.65, and 0.09, respectively. RFI is highly correlated with FI (*r* = 0.89) but essentially unrelated to BW.

RFI regulation is a complex biological process controlled by the coordinated interaction of multiple factors. Prakash proposed that variations in RFI might result from differences in physiological processes of the hen, including feeding behavior, energy homeostasis, nutrient absorption, and metabolic processes, which primarily occur in the central nervous system, metabolic organs, and digestive absorption systems [9]. Metabolites are substances produced by the exchange of materials and energy between an organism and its environment, making them significant for studying metabolic regulation in animals [10]. Notably, blood metabolites can effectively reflect the nutritional status and energy metabolism level of laying hens. Research has found that the systemic concentrations of key blood parameters related to feeding, growth, nutrient allocation, and utilization are potential physiological markers for feed efficiency [11, 12]. For instance, compared to high RFI native chickens, low RFI chickens have lower levels of triiodothyronine (T3), adrenocorticotropic hormone (ACTH), cortisol (COR), and low-density lipoprotein cholesterol (LDL-C), while having higher levels of insulin-like growth factor (IGF-1), serum glucose (GLU), and triglycerides (TG). Thus, it can be demonstrated that IGF-1, T3, COR, and LDL-C can serve as candidate biomarkers for their feed efficiency [13]. Research also indicated that cholesterol could be used as a predictive indicator of feed efficiency [14].

The gut microbiota is a complex community containing trillions of microorganisms. In laying hens, the mucosal interface formed by the single-layer epithelial cells allows microbial metabolites to enter tissue cells and interact, thereby affecting the immune response or causing disease [15]. Studies have shown that disruption of gut microbiota balance can lead to metabolic disorders, affecting carbohydrate, fat, and bile acid metabolic pathways [16]. Additionally, there is an interaction between the poultry gut microbiota and the host's nutritional status, which participate in the metabolism of carbohydrates, fats, proteins, and amino acids in poultry [17]. They play a key role in nutrient digestion and absorption, help develop the immune system, and their community composition and diversity dynamically change with various factor [18]. Given this, the composition of metabolites in the gut or excreta can reflect the efficiency of laying hens in digesting and absorbing feed nutrients.

**Fig. 1:**
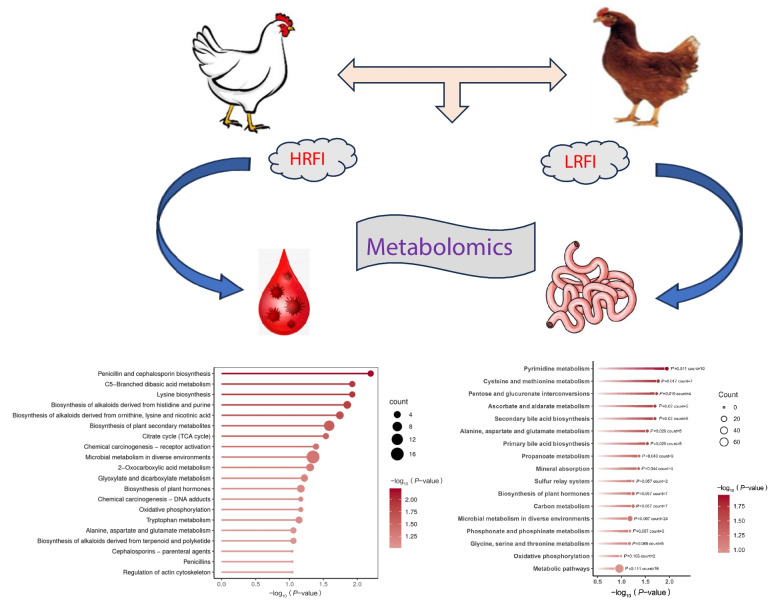
Divergent metabolism and intestinal health underlie variations in residual feed intake

With the advancement of modern molecular biology, metabolomics has become increasingly vital in assessing feed utilization efficiency in laying hens. Previous studies have primarily focused on early to mid-laying stages, leaving a considerable gap regarding the physiological and metabolic changes during the late laying period. This lack of understanding is critical as older hens face unique metabolic challenges that may affect feed efficiency. Our research hypothesizes that the metabolic regulatory networks governing RFI during this crucial phase differ from those observed in younger layers. In this study, we concentrate on blood and excreta analyses to explore phenotypic data, key gene expression, and differences in metabolites between post-laying hens categorized by low RFI and high RFI (Fig. 1). We aim to elucidate these metabolic regulatory mechanisms, thereby contributing to the understanding of how older egg layers may operate under distinct metabolic frameworks compared to their younger counterparts. This research lays a foundation for the future breeding of poultry with high feed efficiency, optimizing performance as hens age.

## Materials and methods

### Experimental design, sample collection and processing

This study utilized a subset of animals and data from a comprehensive, long-term longitudinal investigation reported in our previous work [19]. Briefly, the parent trial involved 1,557 pedigreed Rhode Island Red laying hens (obtained from Beinongda Technology Co., Ltd., Beijing, China) monitored from 27 to 70 weeks of age under standardized conditions (individual cages, 16L:8D photoperiod) and fed a basal diet formulated per national standards [20]. The dietary formulation for laying hens is presented in Table 1. The present study focuses specifically on the measurement period from weeks 67 to 69 of age. From the original population, 1,192 hens remained after excluding statistical outliers (defined as observations for egg weight, body weight, or total feed intake beyond ± 3 standard deviations from the mean). During this 3-week (21d) experimental period, anti-pecking barriers were installed between cages to ensure precise measurement of individual feed intake. Individual body weight was recorded at the start (week 67) and end (week 69) of this period. Eggs were collected, counted, and weighed daily to calculate daily egg mass (DEM, g/d/hen) and total egg production. Daily feed intake (DFI) was measured throughout the 21-d period. Metabolic body weight (MBW, kg) was calculated as BW^0.75^, using the average body weight of the hen over the experimental period. Body weight gain (BWG, g) was the difference between final and initial weights. Daily egg mass (DEM) was calculated as the total egg mass produced over the period divided by 21 d. Feed conversion ratio (FCR) was calculated based on the total feed consumption and total egg mass of the hens during the experimental period. RFI for each hen was calculated as: RFI = TFI − (b₀ + b₁MBW + b₂DEM + b₃BWG), where TFI is the total feed intake from weeks 67–69, and b₀ (intercept), b₁, b₂, and b₃ are the partial regression coefficients derived from a multiple linear regression of TFI on MBW, DEM, and BWG across the population.

**Table 1:** Composition and nutrient contents of the basal diet (as-fed basis), %

Items	Content
Ingredient	
Corn	61.06
Soybean meal	23.80
Soybean oil	0.80
Wheat bran	3.00
Dicalcium phosphate	0.90
Stone powder	9.00
Salt	0.40
Antioxidant	0.04
Antifungal agent	0.05
Premix^1^	0.05
Total	100
Nutrient composition	
AME, MJ/kg	11.13
Crude protein	16.13
Methionine	0.36
Lysine	0.87
Calcium	3.71
Phosphorus	0.58

^1^Vitamin premix provided per kg of diet: vitamin A, 10,000 IU; vitamin D_3_, 3,000 IU; vitamin E, 30 IU; vitamin K_3_, 4 mg; vitamin B_1_, 3 mg; vitamin B_2_, 8 mg; vitamin B_6_, 6 mg, vitamin B_12_, 0.03 mg, niacin, 40 mg, folic acid, 2 mg, biotin, 0.3 mg, pantothenic acid, 18 mg. Mineral premix provided per kg of diet: Mn 100 mg, zinc 100 mg, Fe 65 mg, Cu 9 mg, I 1 mg, Se 0.3 mg

The 21-day period was selected as a standard, sufficient duration for the reliable measurement of feed intake and production traits while minimizing environmental variability. Based on the calculated RFI values from this period, hens were ranked. The seven hens with the lowest RFI values and the seven hens with the highest RFI values were categorically defined as the low RFI (LRFI) and high RFI (HRFI) groups, respectively. This selection was made ensuring no significant difference in average body weight or egg production existed between the two groups at the start of the period, making RFI the sole divergent criterion (Table S1). At 70 weeks of age, hens from these two groups (*n* = 7 per group) were euthanized via rapid cervical dislocation. Blood samples were collected from the wing vein for serum isolation. Segments of the distal ileum and excreta samples were aseptically collected, immediately snap-frozen in liquid nitrogen, and stored at −80 °C for LC–MS metabolomic analysis. For subsequent analyses, a sample size of seven hens per group was designated for the measurement of dietary nutrient utilization, serum biochemical indicators, and physicochemical indices of the ileum. However, due to sample loss, the non-targeted metabolomic analysis of serum and excreta was ultimately performed on six biological replicates per group.

### Measurement of dietary nutrient utilization

After the feeding trial concluded, the experimental hens were placed in metabolic cages, and a 3-d metabolic trial was conducted using the total collection method. An excreta collection tray was placed under the cage to collect and remove feed residues, feathers, and skin flakes, and then excreta were collected, weighed, and mixed with 10% hydrochloric acid (Sinopharm Chemical Reagent Co., Ltd., Shanghai, China) at 10% of excreta weight to fix nitrogen. The excreta samples were stored at −20 °C. Once the experiment ended, the excreta samples collected over 3 d were mixed, dried at 75 °C until constant weight was achieved after re-equilibration for 24 h in a desiccator, thus preparing air-dried samples. The initial moisture content was determined. The samples were milled, sieved through a 40-mesh screen, and stored at −20 °C. Nutrient analyses were conducted for both the diet and excreta samples according to corresponding national standards. These analyses included crude fat [21], crude protein [22], crude fiber [23], dry matter content [24], acid-insoluble ash with hydrochloric acid [25], and total phosphorus [26]and total calcium [27]. The determination of these indicators was conducted in accordance with the national standards of the People's Republic of China. The apparent nutrient utilization percentages were then calculated using the following formula: Apparent nutrient utilization (%) = [100 − (*a* × *c*)/(*b* × *d*)] × 100, where *a* is the nutrient content in excreta, *b* is the nutrient content in feed, *c* is the acid-insoluble ash content in feed, and *d* is the acid-insoluble ash content in excreta.

### Non-targeted metabolomics of excreta

After the feeding trial concluded, excreta samples (80 mg) were flash-frozen in liquid nitrogen, homogenized in H₂O (200 μL) with ceramic beads, extracted using methanol/acetonitrile (1:1, 800 μL) (Merck, Darmstadt, Germany), centrifuged (14,000 × *g*, 15 min, 4 °C), dried, and reconstituted similarly. UPLC-Q-TOF/MS analysis (Agilent 1290 Infinity LC coupled to AB Sciex TripleTOF 6600) was performed at Shanghai Applied Protein Technology Co., Ltd. (Shanghai, China).

### Measurement of serum biochemical indicators

After the feeding trial concluded, 200 μL serum from each hen was collected and sent to Beijing Jinhaikeyu Biotechnology Development Co., Ltd. They used the company's reagent kits and a fully automated biochemical analyzer (Kehua ZY KHB-1280) to measure the contents of free fatty acid (FFA), orexin, total bile acid (TBA), urea (URE), cholesterol (CHO), malondialdehyde (MDA), superoxide dismutase (SOD), catalase (CAT), total antioxidant capacity (T-AOC), and glutathione peroxidase (GSH-PX) in the serum.

### Non-targeted metabolomics of serum

After the feeding trial concluded, fasting blood in EDTA tubes was centrifuged, and the plasma was stored at –80 °C. The plasma was then thawed and mixed with cold methanol/acetonitrile (1:1) for deproteinization. After centrifugation, the supernatant was dried and redissolved in acetonitrile/water (1:1) for analysis using Agilent 1290 Infinity UHPLC (Agilent Technologies, Santa Clara, CA, USA) coupled to AB Sciex TripleTOF 6600 (AB Sciex, Framingham, MA, USA). HILIC separation used an ACQUIY UPLC BEH column with ammonium acetate/hydroxide-acetonitrile mobile phase, and RPLC used an ACQUIY UPLC HSS T3 column with acid/fluoride-containing mobile phases, both with specific gradients. ESI conditions included gas flows, temperature, and voltage; MS/MS covered relevant *m*/*z* ranges and parameters. Data processed via ProteoWizard and XCMS, metabolites identified against in-house standards, with KNN for missing values and normalization.

### Reverse transcription quantitative real-time PCR (RT-qPCR) of ileum

After the hens were slaughtered ileum tissue samples (50–100 mg) were collected. The samples were stripped of their mucosal layer and RNA was then extracted from the underlying intestinal tissue using Trizol (GenScript, Beijing, China). After extracting the RNA its quality and concentration were assessed using an Epoch2 spectrophotometer (BioTek USA). The total RNA was reverse-transcribed using the BeyoRT™ II cDNA Synthesis Kit (Beyotime Biotechnology, Shanghai, China) and stored at −20 °C for later use. RT-qPCR was performed using the BeyoFast™ SYBR Green qPCR Mix (Beyotime Biotechnology, Shanghai, China) on the Applied BiosystemsQuant Studio 7500 system (Thermo Fisher Scientific, Waltham, MA, USA) for fluorescence detection of the relative mRNA abundance of target genes. The primers for the target genes were designed using Primer Premier 5.0 software, while the primer sequence for the internal reference gene (β-actin) was obtained from established literature. The design of primers followed general primer design principles, with a focus on high specificity. The specificity of all designed primers was verified using the NCBI Primer-BLAST tool. To prevent potential genomic DNA amplification from affecting the quantitative results, primer pairs were designed to span exon-exon junctions where possible. The expected amplicon sizes were set between 100 to 300 base pairs. The primer sequences used in this study are listed in Table 2; these were synthesized by Shanghai Sangon Biotech Co., Ltd. (Shanghai, China). The relative expression levels of the target gene mRNA were calculated using the 2^−ΔΔCt^ method.

**Table 2:** Primer sequences for RT-qPCR analysis

Gene	Primer sequences (5′→3′)	Accession No.
*β-actin*	F: GAGAAATTGTGCGTGACATCA	L08165
	R: CCTGAACCTCTCATTGCCA	
*Occludin*	F: ACGGCAGCACCTACCTCAA	NM_205128.1
	R: GGGCGAAGAAGCAGATGAG	
*Claudin-1*	F: CATACTCCTGGGTCTGGTTGGT	NM_001013611.2
	R: GACAGCCATCCGCATCTTCT	
*ZO-1*	F: CTTCAGGTGTTTCTCTTCCTCCTC	XM_413773
	R: CTGTGGTTTCATGGCTGGATC	
*TNF-α*	F: GAGCGTTGACTTGGCTGTC	NM_204267
	R: AAGCAACAACCAGCTATGCAC	
*IL-10*	F: CGCTGTCACCGCTTCTTCA	NM_001004414.4
	R: TCCCGTTCTCATCCATCTTCTC	
*IL-6*	F: GCTCGCCGGCTTCGA	AJ250838
	R: GGTAGGTCTGAAAGGCGAACAG	

### Western blot analyses of ileum tissue

Ileum tissues were homogenized in ice-cold RIPA lysis buffer (Beyotime Biotechnology, Shanghai, China) using a cryogenic grinder. The homogenate was centrifuged at 13,000 × *g *for 15 min at 4 °C to collect the supernatant, and protein concentration was determined using the BCA assay (Beyotime Biotechnology, Shanghai, China). Total protein was mixed with 5 × SDS-PAGE loading buffer (Beyotime Biotechnology, Shanghai, China), denatured at 95 °C for 10 min, and stored at −80 °C until use. Proteins were resolved on 10% SDS-PAGE gels and transferred to PVDF membranes using a semi-dry transfer system. Membranes were blocked with 5% non-fat milk in TBST for 1 h. Primary antibodies against ZO-1​​ (Bioss, Beijing, China) and ​​Claudin 1 (ABclonal, Wuhan, China) were diluted 1:1,000 in TBST containing 5% BSA and incubated with membranes overnight at 4 °C. After three washes with TBST, membranes were incubated with HRP-conjugated goat anti-rabbit secondary antibody (1:5,000 dilution) for 1 h. ​​β-Actin​​ (Bioss, bs-0061R, 1:1,000 dilution) was used as a loading control. Protein bands were visualized using enhanced chemiluminescence (ECL) substrate and imaged on a ChemiDoc™ system (Bio-Rad). Band intensities were quantified using Image Lab™ software, normalized to β-actin, and statistically analyzed.

### Data analysis

Statistical analysis was performed using SPSS Statistics 25.0 software (IBM Corp., Armonk, NY, USA). All data were first tested for normality of distribution. For data meeting the assumption of normality, differences between the two groups were analyzed using an unpaired Student’s *t*-test. For data that did not follow a normal distribution, the non-parametric Mann–Whitney U test was applied. Significant differences between groups are indicated as* P* < 0.05 and highly significant differences as *P* < 0.01. Data are presented as the mean ± standard error of the mean (SEM). Graphs were generated using GraphPad Prism 9.0 (GraphPad Software, San Diego, CA, USA).

## Results

### Effects of different RFI on nutrient utilization in laying hens

**Fig. 2:**
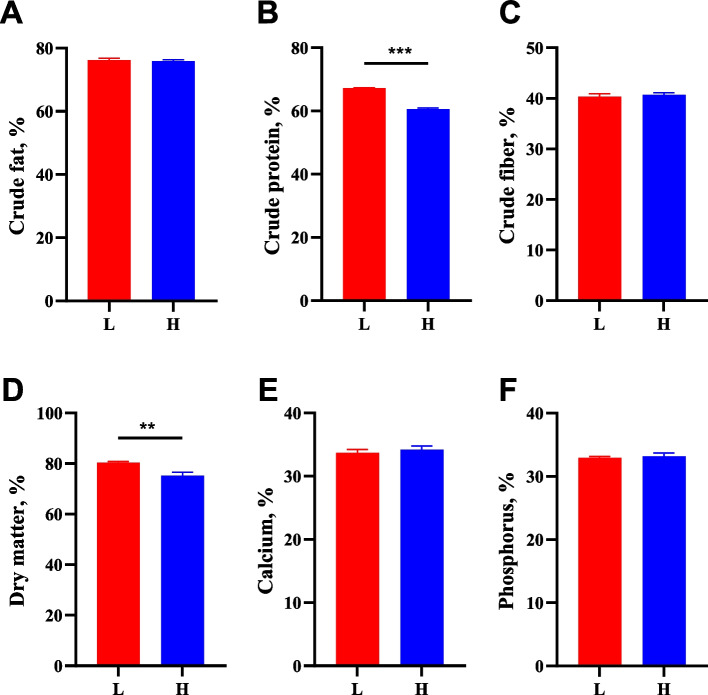
Different RFI groups on nutrient utilization efficiency. **A** Crude fat content. **B** Crude protein content. **C** Crude fiber content. **D** Dry matter content. **E** Total calcium content. **F** Total phosphorus content. Data are represented as the means ± SEM (*n* = 7), ^*^P ≤ 0.05, ^**^P ≤ 0.01

As shown in Fig. 2, compared to the HRFI group, the LRFI group had a significant increase in apparent crude protein utilization and dry matter apparent utilization (*P* < 0.05). However, there were no significant differences between the two groups in the apparent utilization of crude fat, crude fiber, calcium, and phosphorus (*P* > 0.05).

### Effects of different RFI on serum biochemical indicators in laying hens

**Fig. 3:**
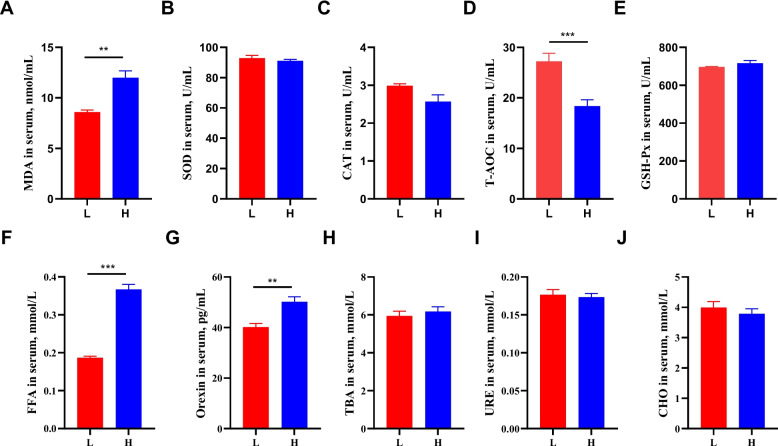
Different RFI on serum physiochemical indices. **A** Malondialdehyde content. **B** Superoxide dismutase content. **C** Catalase content. **D** Total antioxidant capacity. **E** Glutathione peroxidase activities. **F** Free fatty acids contents. **G** Orexin contents. **H** Total bile acids contents. **I** Urea contents. **J** Cholesterol contents; Data are represented as the means ± SEM (*n* = 7), ^*^P ≤ 0.05, ^**^P ≤ 0.01

 In terms of serum antioxidant indicators, the MDA content in the HRFI group was significantly higher than that in the LRFI group (*P* < 0.05), while the T-AOC content was significantly lower in the HRFI group (*P* < 0.05). There were no significant differences between the two groups in the contents of SOD, CAT, and GSH-PX (*P* > 0.05) (Fig. 3A–E). In terms of serum glucose lipid metabolism and feeding-related hormone indicators, the contents of orexin and FFA in the HRFI group were significantly higher than those in the LRFI group (*P* < 0.05), while there were no significant differences between the two groups in the contents of TBA, URE, and CHO (*P* > 0.05) (Fig. 3F–J).

### Effects of different RFI on serum metabolites in laying hens

**Fig. 4:**
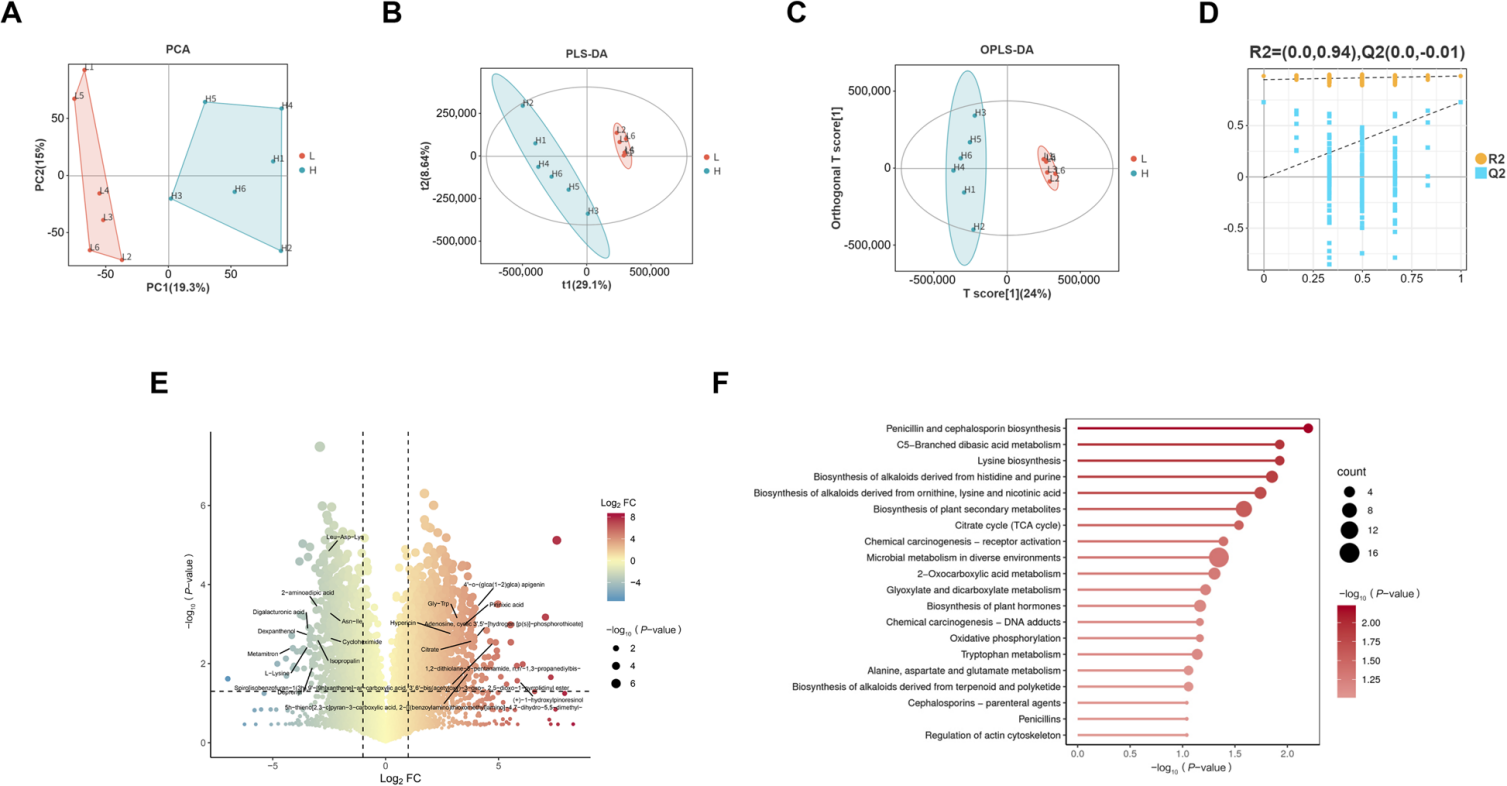
Multivariate statistical analysis of serum metabolomics in the LRFI and HRFI groups (*n* = 6 per group). **A** PCA analysis (combination of positive ion and negative ion). **B** PLS-DA analysis (combination of positive ion and negative ion). **C** OPLS-DA analysis (combination of positive ion and negative ion). **D** Permutation test plot of metabolomics. **E** Volcano plot (combined + ve and – ve ions); **F** KEGG pathway enrichment analysis (combined + ve and – ve ions)

**Fig. 5:**
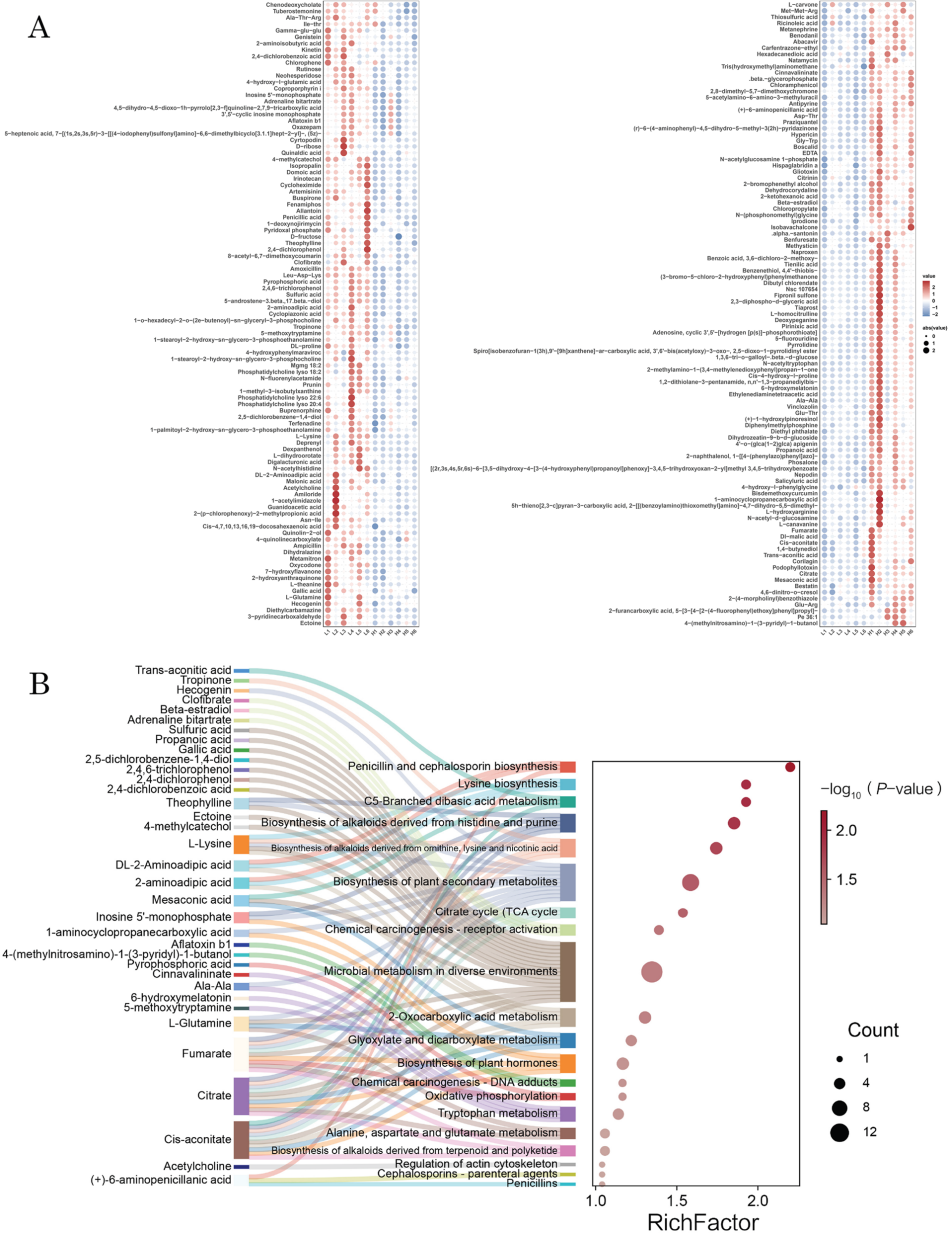
Differential metabolite profiling and functional annotation in the LRFI and HRFI groups (*n* = 6 per group). **A** DEMs expression in cluster heatmap. **B** Sankey diagram combined with KEGG *P*-value plot

In this study, the HRFI and LRFI groups demonstrated clear clustering, indicating alterations in endogenous small-molecule metabolites and significant differences in serum metabolites between the two groups (Fig. 4A–C). As shown in Fig. 4D, the regression line of Q2 in the permutation test intersected below zero on the *y*-axis, and the rightmost points had R2 values higher than those on the left side or the original Q2 points. This suggests that there is no overfitting and the model validation is valid. The volcano plot showed that there were 198 metabolites with significant differences between the two groups (*P* < 0.05, VIP > 1, Fold change > 2.0 or < 0.5), with 101 upregulated and 97 downregulated metabolites (Fig. 4E). To better reflect the distributional differences of metabolites, hierarchical clustering analysis was performed on these 198 differentially abundant metabolites based on VIP values (Fig. 5A). KEGG metabolic pathways are typically divided into seven major categories, including Metabolism, Genetic Information Processing, Environmental Information Processing, Cellular Processes, Organismal Systems, Human Diseases, and Drug Development. Afterward, we conducted KEGG pathway analysis on the differentially abundant metabolites in the serum of both groups. Figure 4F show the top 20 metabolic pathways enriched by differentially abundant metabolites between the two groups. We found that the metabolites are primarily enriched in pathways such as Penicillin and cephalosporin biosynthesis, Lysine biosynthesis, Citrate cycle (TCA cycle), Microbial metabolism in diverse environments, and 2-Oxocarboxylic acid metabolism. The KEGG pathway enrichment analysis of differentially abundant metabolites was visualized using a chord diagram (metabolite-pathway associations) and a bubble plot (pathway significance). Key findings included trans-aconitic acid and tropinone being linked to Penicillin and cephalosporin biosynthesis, while citrate and fumarate were associated with the TCA cycle. The bubble plot identified Penicillin and cephalosporin biosynthesis as the most significant pathway, concurrently enriching energy metabolism pathways (e.g., Oxidative phosphorylation) and amino acid metabolic pathways, each involving multiple differentially abundant metabolites (Fig. 5B). Metabolites significantly enriched in these pathways, such as L-lysine, L-glutamine, *cis*-aconitate, inosine 5'-monophosphate, citrate, fumarate, and propanoic acid, showed significant differences between the two groups' serum samples, with all being significantly increased in the LRFI group serum and are of particular interest for further investigation.

### Effects of different RFI on gut health in laying hens

**Fig. 6:**
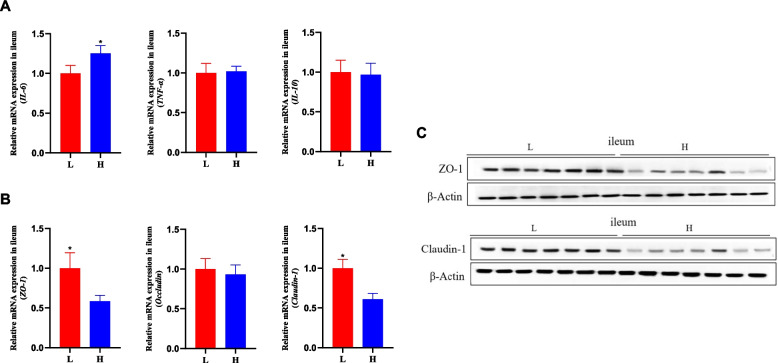
Different RFI groups on physicochemical indices of ileum. **A** Relative expression of genes related to intestinal immunological indices. **B** Relative expression of genes related to intestinal barrier indices. **C** Western blot of intestinal barrier indices; Data are represented as the means ± SEM (*n* = 7), ^*^P ≤ 0.05

To gain a deeper understanding of the mechanisms underlying the differences in gut damage caused by different RFI levels, we tested for indicators related to gut barrier damage and immunity in both groups of hens. The results showed that at the mRNA level, compared to the HRFI group, *Claudin-1* and *ZO-1* were significantly upregulated (*P* < 0.05) in the ileum tissue of the LRFI group, while *IL-6* was significantly downregulated (*P* < 0.05). However, there was no significant difference in the mRNA expression of *TNF-α*, *IL-10*, and *Occludin* between the two groups of hens (*P* > 0.05) (Fig. 6A and B). At the protein level, the protein expression levels of Claudin-1 and ZO-1 in the ileum tissue of the LRFI group were significantly higher than those in the HRFI group (*P* < 0.05) (Fig. 6C).

### Effects of different RFI on excreta metabolites in laying hens

**Fig. 7:**
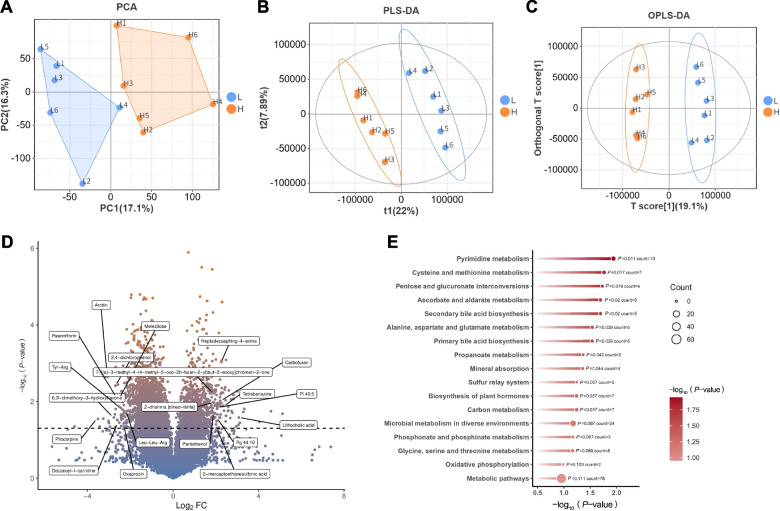

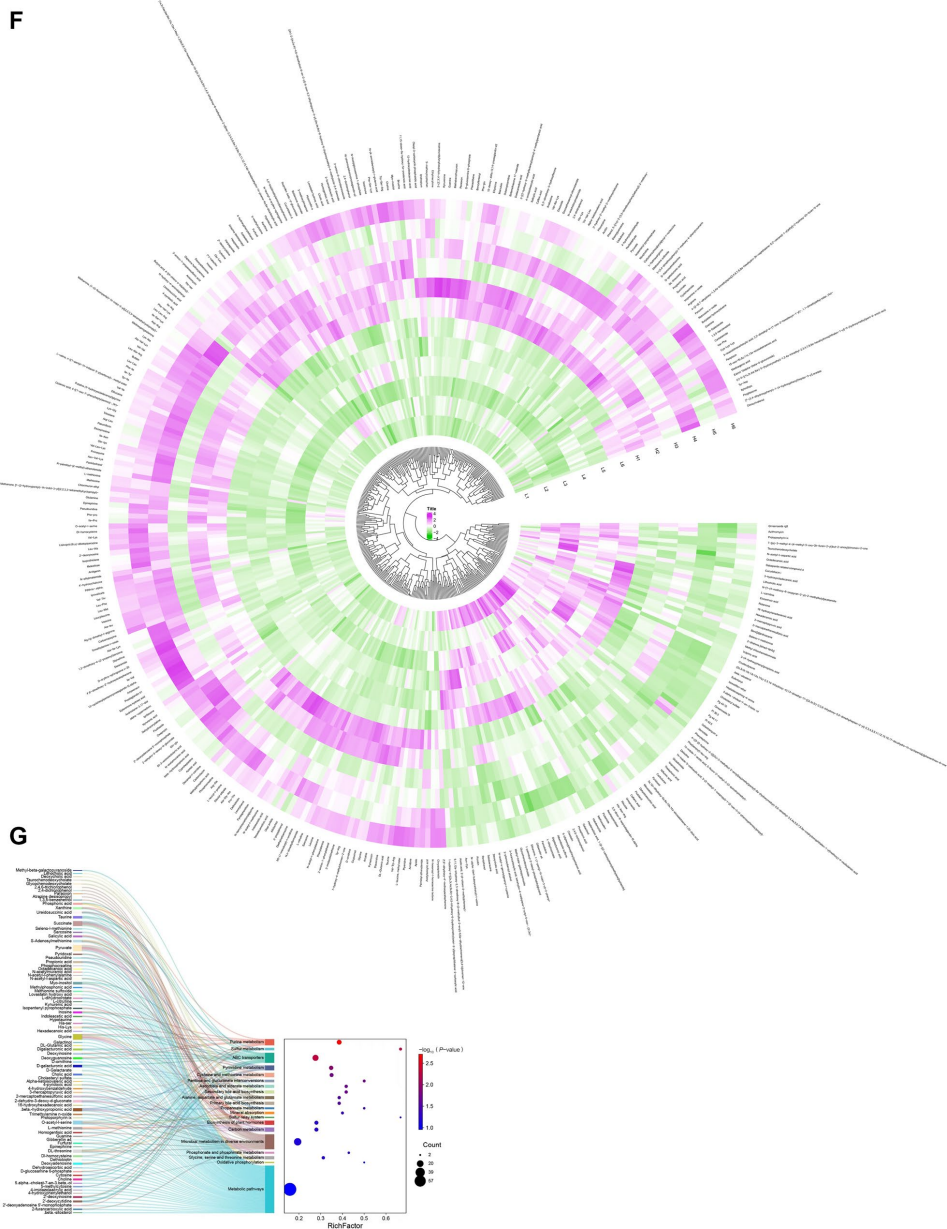
Untargeted metabolomics analysis of the poultry excreta in the LRFI and HRFI groups (*n* = 6 per group). **A** PCA analysis (combination of positive ion and negative ion). **B** PLS-DA analysis (combination of positive ion and negative ion). **C** OPLS-DA analysis (combination of positive ion and negative ion). **D** Volcano plot (combined + ve and – ve ions). **E** KEGG pathway enrichment analysis (combined + ve and – ve ions). **F** DEMs expression in cluster heatmap. **G** Sankey diagram combined with KEGG *P*-value plot

This experiment used LC–MS technology to detect metabolites in the excreta of LRFI and HRFI layers. After pre-processing, PCA, PLS-DA, and OPLS-DA analyses were performed on the data, and Student’s *t*-test results (confidence level > 0.95) indicated significant differences in PLS-DA, OPLS-DA scores, and differential metabolites clustering analysis between the LRFI and HRFI groups, suggesting substantial differences in metabolite levels between the two groups (Fig. 7A–C). Volcano plots visually presented the contributions of potential biomarkers, with each point representing one metabolite (*P* < 0.05, VIP > 1, Fold change > 2.0 or < 0.5). Analysis resulted in the identification of 357 known differentially abundant metabolites in excreta samples from the two groups under both positive and negative ion modes, among which 92 metabolites were significantly upregulated and 265 were significantly downregulated (Fig. 7D). KEGG pathway enrichment analysis of the screened differentially abundant metabolites revealed significant enrichment in pathways such as Pyrimidine metabolism, Cysteine and methionine metabolism, Pentose and glucuronate interconversions, Ascorbate and aldarate metabolism, Secondary bile acid biosynthesis, Alanine, aspartate and glutamate metabolism (Fig. 7E). Visualizations of KEGG pathway enrichment for differential metabolites were achieved using a chord diagram (metabolite-pathway connections) and a bubble plot. In the chord diagram, glycine and taurine were mapped to Glycine metabolism, while hexadecanoic acid was associated with Oxidative phosphorylation. The bubble plot highlighted prominent enrichment in Glycine metabolism and Purine metabolism, along with pathways involved in amino acids (e.g., glycine, serine and threonine metabolism) and energy metabolism (Oxidative phosphorylation), each containing multiple differentially abundant metabolites. Metabolites significantly enriched in these pathways, such as glycine, pyruvate, taurine, succinate, cholic acid, glycochenodeoxycholate, and taurochenodeoxycholate, exhibited significant distinctions between the two layers' sera and appeared repeatedly among the above-enriched metabolic pathways, and they are also key metabolites of future focus (Fig. 7F and G).

## Discussion

Currently, in poultry farming, feed efficiency (FE) is mainly measured by FCR and RFI [28]. FCR is a trait calculated through a ratio, and selecting for FCR alone might lead to issues where chickens have the same FCR but significant differences in feed intake and weight gain [9]. RFI, as another indicator of feed utilization efficiency, is defined as the difference between actual feed intake and the predicted feed intake based on the chicken's metabolic weight, weight gain, and egg production [29]. Previous research in chickens, both layers and broilers, as well as in cattle, found that selective breeding based on RFI does not negatively affect animal production performance [30, 31]. Combined with our previous research results, which indicate that RFI is independent of growth traits in post-laying hens [19], we selected seven hens with the highest and lowest RFI values from each group, under conditions of consistent body weight and egg production, defining them as the LRFI and HRFI groups, respectively.

Apparent nutrient utilization primarily reflects an animal's absorption of nutrients from the diet [32]. The absorption of nutrients by laying hens directly impacts their production performance and egg quality, making apparent nutrient utilization an important indicator for assessing how effectively laying hens absorb and utilize dietary nutrients [33]. Our study results show that LRFI hens exhibit higher apparent utilization rates for crude protein and dry matter. Research has found that improving the apparent utilization of crude protein and dry matter has a significant positive impact on feed efficiency in late-laying hens [1]. Higher apparent utilization of crude protein means that hens can absorb and utilize dietary protein more effectively, promoting protein synthesis and enhancing the efficiency of protein use [34]. This directly contributes to improving egg production performance and quality while extending the laying cycle. Secondly, increased apparent utilization of dry matter indicates that hens can better digest and absorb nutrients from feed, reducing feed waste [35]. This not only enhances feed conversion rates but also helps in lowering feeding costs. From these findings, we can preliminarily conclude that, compared to HRFI hens, LRFI hens have a stronger ability to absorb nutrients and higher nutrient utilization efficiency.

Studies have indicated that systematic changes in the concentrations of key blood parameters related to feeding, growth, nutrient allocation, and metabolic utilization can serve as potential physiological markers of feed efficiency [11, 36]. Additionally, the concentrations of FFA, appetite-related hormones, and antioxidative enzyme activities in serum can reflect the relationship between lipid and energy metabolism, and the level of immune metabolism [37]. Previous research found that compared to hens with high RFI, those with low RFI showed lower serum glucose levels and higher immunoglobulin levels [14, 38]. Furthermore, Sierżant et al. [39]discovered that pigs with high feed efficiency were less susceptible to oxidative stress during production compared to those with low feed efficiency. In this study, we found that compared to the HRFI group, hens in the LRFI group had significantly higher serum T-AOC, while levels of MDA, FFA, and orexin were significantly reduced. Studies have demonstrated that FFA, as a crucial component of energy metabolism, can influence feeding behavior by regulating hunger and satiety [40]. Elevated FFA levels may stimulate appetite and increase feed intake. Moreover, FFA is involved in lipid metabolism and can exacerbate fat accumulation in the liver, causing metabolic burden [41]. Orexin is a neuropeptide that functions in the central nervous system; its release enhances appetite and promotes feeding behavior [42]. The antioxidative capacity of an animal’s defense system also indicates its health status [43]. MDA, a product of lipid peroxidation. Increased MDA may affect intestinal health and function, leading to reduced nutrient absorption [44]. Based on these experimental findings, we can infer that excessive feed consumption in HRFI group hens may lead to metabolic disorders, thereby causing cellular oxidative stress.

Additionally, we utilized LC–MS to detect changes in serum metabolites of hens from both groups. The results indicated that the differentially abundant metabolites were primarily enriched in pathways such as Lysine biosynthesis, TCA cycle, Microbial metabolism in diverse environments, and 2-Oxocarboxylic acid metabolism. These pathways are primarily involved in energy metabolism, amino acid metabolism and participate in processes such as lipid metabolism. Among these enriched metabolites, those closely related to avian digestion, utilization, and feed intake have been confirmed to have a close relationship with RFI [45, 46]. Research has found differences in energy metabolism between LRFI pigs and HRFI pigs. LRFI pigs displayed higher energy efficiency, lower feed intake, and reduced energy consumption from physical activity [47]. Duarte's lipidomics analysis in cattle found higher fatty acid (FA) and triglyceride (TG) levels in animals with high RFI compared to those with medium and low RFI levels [48]. This suggests that animals with low RFI might have an increased demand for fat energy sources, while those with high RFI exhibit increased fat deposition. In our study, metabolites such as L-lysine, L-glutamine, *cis*-aconitate, inosine 5'-monophosphate, citrate, fumarate, and propanoic acid were significantly upregulated in the LRFI group. Current research on the regulatory mechanisms of L-lysine on chicken feed intake varies. Some studies have suggested that high concentrations of L-lysine may lead to competition and imbalance of other amino acids, thereby inhibiting overall feed appetite [49]. However, other studies have found that L-lysine, as an essential amino acid, can promote the absorption and utilization of other amino acids in chickens. When dietary lysine levels are adequate, it can stimulate feed intake and increase overall consumption [50]. L-Glutamine is an important energy source for intestinal epithelial cells and can promote gut repair and health. Increased levels of L-glutamine can enhance chickens' absorption and utilization of feed nutrients, thereby improving feed utilization efficiency to some extent [51]. Inosine 5'-monophosphate, citrate, and fumarate are key intermediates involved in TCA cycle, playing roles in ATP production [52]. Increasing their concentration can accelerate ATP synthesis, allowing chickens to generate more energy while reducing the need for additional feed consumption. Additionally, increased levels of citrate and fumarate may optimize energy use efficiency by regulating metabolic pathways, affecting fatty acid oxidation and glucose metabolism [53]. This optimization helps maintain physiological balance, prevent energy deficiency due to insufficient feed intake. Propionic acid, as a short-chain fatty acid, is quickly absorbed during digestion and can inhibit appetite by activating specific receptors [54]. This might cause hens to feel satiated sooner after feeding, thus reducing feed intake. Furthermore, Propionic acid can enhance energy availability, enabling a decrease in additional feed demand when energy needs are met, naturally reducing feed intake [55]. In summary, compared to HRFI hens, LRFI hens have accelerated energy metabolism, maintaining energy demands during production and consequently reducing feed intake while improving feed utilization efficiency.

The ileum is the primary site for the absorption of amino acids, short-chain fatty acids, and other essential nutrients [56]. A healthy ileal structure supports the stability of beneficial bacteria, suppresses harmful bacterial growth, and enhances the intestinal barrier function [57]. Previous studies have found that groups with lower RFI in livestock often exhibit more robust immune and mechanical barrier functions in the gut [58]. As the gut is the direct interface between the body and the external environment, it plays a vital role in maintaining bodily health [59]. Gut inflammation responses are closely linked to the expression levels of pro-inflammatory and anti-inflammatory factors, whose interactions maintain immune homeostasis in the gut. Increased intestinal epithelial permeability can lead to contact between luminal pathogens, toxins, antigens, and immune cells in the lamina propria, triggering and exacerbating gut inflammation [60, 61]. Therefore, we examined indicators related to ileal tissue immunity and intestinal mechanical barrier function. Our findings indicate that the pro-inflammatory factor (*IL-6*) was significantly downregulated in the LRFI group; tight junction proteins (*Occludin*,* ZO-1*) were significantly upregulated in the LRFI group. Studies have shown that IL-6 production is closely related to gut health. Elevated *IL-6* can trigger gut inflammation, affecting nutrient absorption and reducing feed utilization efficiency [62]. Occludin, composed of four transmembrane domains, can relocate to different extracellular positions, thereby altering epithelial cell permeability. During barrier dysfunction, Occludin frequently moves from tight junctions into cytoplasmic vesicles, a phenomenon triggered by various stimuli [63]. Cani et al. [64] demonstrated that Occludin expression is inversely correlated with the translocation of FITC-dextran from the gastrointestinal tract into the bloodstream. ZO proteins include ZO-1, ZO-2, and ZO-3. Compared to ZO-2 and ZO-3, ZO-1 plays a primary role in forming TJs in epithelial cells. It connects transmembrane proteins to the cytoskeleton and is considered a critical component of tight junctions [65]. Based on these indicators, we observed that compared to the HRFI group, hens in the LRFI group displayed a healthier gut state. This contributes to an enhanced ability of hens to absorb nutrients, subsequently reducing feed intake.

This study, through excreta metabolomics analysis, revealed that there are significant differences in the composition of gut content metabolites between LRFI and HRFI laying hens during the late laying period. These metabolic differences are primarily enriched in pathways related to pyrimidine metabolism, cysteine and methionine metabolism, secondary bile acid biosynthesis, amino acid metabolism, and energy metabolism. These findings suggest that metabolic characteristics of gut microbial-host interactions might be an essential mechanism for feed efficiency differences between the two groups. Examining the mechanisms underlying these differences, significant changes in excreta metabolites first reflect the specificity of gut microbial metabolic activity and host-microbial interactions [66]. For instance, enrichment in the secondary bile acid biosynthesis pathway may relate to the gut microbiota's ability to convert bile acids. Secondary bile acids are involved not only in fat digestion and absorption but also regulate energy metabolism by activating signaling pathways like farnesoid X receptor (FXR) [67, 68]. Differences in their levels may directly impact the host's efficiency of utilizing lipids in feed, leading to divergence in energy acquisition between LRFI and HRFI hens. Additionally, enrichment in amino acid metabolism pathways is another key difference. For example, as a vital one-carbon unit donor, variations in glycine levels might affect the proliferation and repair of intestinal mucosal cells, thereby altering nutrient absorption efficiency [69]. Taurine, acting as a bile acid binder, may synergistically influence lipid absorption with secondary bile acid metabolism, indirectly affecting feed conversion efficiency [70]. On the energy metabolism level, as a key intermediate product in the tricarboxylic acid cycle, levels of succinic acid may indicate gut microbial capacity for energy production, while pyruvic acid acts as an important hub in carbohydrate and lipid metabolism [71]. Their differences may impact the host's efficiency in breaking down and utilizing carbohydrates and fats from feed, contributing to regulation of feed efficiency. Additionally, enrichment in purine and pyrimidine metabolism may reflect increased proliferative activity of the gut microbiota, thereby influencing host competition for and utilization of nutrients [72]. With respect to feed intake regulation, excreta metabolites may act through the "gut-brain axis" or local intestinal signaling pathways. In summary, the observed excreta metabolic differences between LRFI and HRFI laying hens during the late laying period primarily arise from divergent microbial metabolic activities involving bile acids, amino acids, and energy substrates. These differential metabolites participate in regulating nutrient absorption efficiency, energy metabolism balance, and appetite signaling pathways, collectively contributing to variations in feed efficiency and intake regulation.

## Conclusions

In summary, this study establishes RFI as a key indicator of feed efficiency in late-laying hens. Hens with LRFI exhibited superior feed efficiency, underpinned by enhanced nutrient utilization, greater antioxidant capacity, and better intestinal health compared to their HRFI counterparts. Metabolomic analyses revealed that these advantages are associated with distinct metabolic profiles, particularly in amino acid and energy metabolism. Crucially, our findings in this later production phase differ from observations in younger hens; the superior efficiency of LRFI hens here appears driven by a concerted metabolic adaptation to maintain homeostasis and nutrient partitioning after peak lay, rather than by mechanisms primarily supporting rapid growth and peak egg output. This suggests a distinct, efficiency-sustaining mechanism operational in older, high-performing layers.

## Supplementary Information

Additional file 1: Table S1. Production performance of laying hens.

Additional file 2: Fig. S1. Western blot detection of tight junction-associated proteins.

## Data Availability

All data generated or analyzed during this study are available from the corresponding author upon reasonable request.

## Abbreviations

CAT: Catalase
CHO: Cholesterol
FFA: Free fatty acid
GSH-Px: Glutathione peroxidase
IL-10: Interleukin-10
IL-6: Interleukin-6
MDA: Malondialdehyde
SOD: Superoxide dismutase
T-AOC: Total antioxidant capacity
TBA: Total bile acid
TNF-α: Tumor necrosis factor-α
URE: Urea
ZO-1: Zonula occludens-1
