# Supplementary material for: Residual feed intake in laying hens during the late laying period: associations with nutrient utilization, antioxidant capacity, and gut barrier function
Source: J Anim Sci Biotechnol. 2026 Jul 28;17:157. doi: 10.1186/s40104-026-01426-7 (PMC13410610; doi:10.1186/s40104-026-01426-7)
Supplement: Supplementary file 1 — Additional file 1: Table S1. Production performance of laying hens. [file 40104_2026_1426_MOESM1_ESM.docx]

**Table S1** Production performance of laying hens

| **Traits^1^** | **LRFI** | **HRFI** | ***P*-value** |
| --- | --- | --- | --- |
| EN | 313.10±6.05 | 313.50±5.51 | 0.93 |
| AEW | 58.38±1.98 | 58.26±2.01 | 0.94 |
| BW | 3038.22±45.22 | 3099.23±67.09 | 0.78 |
| FI | 2208.75±40.86^b^ | 2500.37±33.86^a^ | 0.02 |
| FCR | 2.31±0.28^b^ | 2.78±0.39^a^ | 0.02 |
| RFI | -132.94±15.38^b^ | 105.2±17.96^a^ | < 0.001 |

^1^ EN, total egg number at 70 weeks of age; AEW, average egg weight from 67 to 69 weeks of age; BW, Body weight at 70 weeks of age; FI, feed intake from 67 to 69 weeks of age; FCR, feed conversion ratio from 67 to 69 weeks of age; RFI, residual feed intake from 67 to 69 weeks of age. Values are presented as the mean and SEM (*n* = 7, 67-69 weeks of age), and different letters indicate substantial variations (*P* < 0.05)
