# Supplementary figures and images for: Residual feed intake in laying hens during the late laying period: associations with nutrient utilization, antioxidant capacity, and gut barrier function
Source: J Anim Sci Biotechnol. 2026 Jul 28;17:157. doi: 10.1186/s40104-026-01426-7 (PMC13410610; doi:10.1186/s40104-026-01426-7)

**Supplementary Figures**


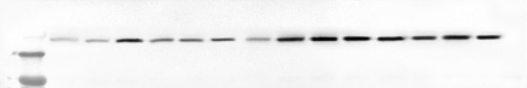


ZO-1


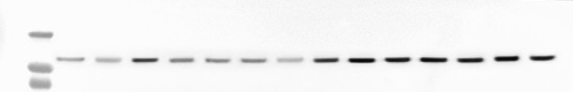


Clauding-1


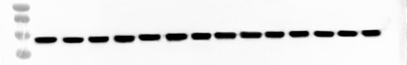


β-actin


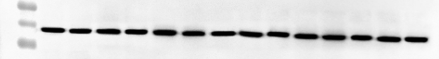


β-actin

Supplement: Supplementary file 2 — Additional file 2: Fig. S1. Western blot detection of tight junction-associated proteins. [file 40104_2026_1426_MOESM2_ESM.docx]
